# Impact of Adiposity and Fat Distribution, Rather Than Obesity, on Antibodies as an Illustration of Weight-Loss-Independent Exercise Benefits

**DOI:** 10.3390/medicines8100057

**Published:** 2021-10-08

**Authors:** Abdelaziz Ghanemi, Mayumi Yoshioka, Jonny St-Amand

**Affiliations:** 1 Functional Genomics Laboratory, Endocrinology and Nephrology Axis, CHU de Québec-Université Laval Research Center, Québec, QC G1V 4G2, Canada; Abdelaziz.Ghanemi@crchudequebec.ulaval.ca (A.G.); mayumi.yoshioka@crchudequebec.ulaval.ca (M.Y.); 2 Department of Molecular Medicine, Faculty of Medicine, Laval University, Quebec, QC G1V 0A6, Canada

**Keywords:** obesity, adiposity, antibodies, immunity

## Abstract

Obesity represents a risk factor for a variety of diseases because of its inflammatory component, among other biological patterns. Recently, with the ongoing COVID-19 crisis, a special focus has been put on obesity as a status in which antibody production, among other immune functions, is impaired, which would impact both disease pathogenesis and vaccine efficacy. Within this piece of writing, we illustrate that such patterns would be due to the increased adiposity and fat distribution pattern rather than obesity (as defined by the body mass index) itself. Within this context, we also highlight the importance of the weight-loss-independent effects of exercise.

Obesity is one of most challenging health problems for the modern medicine and therapeutic research [1,2]. The main pattern that makes obesity challenging is that, once established, it is hard to reverse, probably because the new “set up” of the biological reference of body weight and adiposity as neuroendocrine adaptation changes with a broken energy homeostasis [3,4]. The current ongoing coronavirus disease 2019 (COVID-19) pandemic could worsen the obesity pandemic, which would negatively impact the development of this COVID-19 crisis [5,6], especially with the impact that the measures imposed by governments might have on immunity [7]. Therefore, it is of high importance to understand how obesity and adiposity impact the immunity and more specifically antibodies production and function. This is because vaccine-induced antibodies represent the best shot we have to end this pandemic.

Antibodies represent an important mediator and factor of the immune system [8]. On the other hand, obesity represents a status in which different biological and homeostatic processes, such as regeneration [9], energy balance [4] and neuroendocrine factors [3], are impaired or impacted. Within this context, we would like to put a spotlight on selected consequences and impacts obesity and adiposity have on antibody patterns in order to explain some immunological specificities reported in obese patients. Obesity is defined by an abnormal fat accumulation usually as a result of an unhealthy lifestyle that increases the energy intake to more than the energy expenditure [1,4], leading to a variety of health consequences [10,11] with increased impacts [5].

Regarding obesity-related antibody patterns, numerous results reflect the impacts obesity has on antibody properties. For instance, adaptive immune response to influenza virus is impaired during obesity [12], innate and adaptive immune responses against influenza are delayed in obese patient [13] and obesity was suggested to decline influenza antibody titers following influenza vaccination [14] as well as reduce vaccine efficacy [15] with poor vaccine immunogenicity [16]. Similarly, lower COVID-19 mRNA vaccine-induced antibody titers have been associated with central obesity [17] and severe acute respiratory syndrome corona Virus-2 IgG antibodies negatively correlate with body mass index in COVID-19 patients. This is important in the current pandemic context with the vaccination efforts aiming to end this global health crisis. Furthermore, one key concept in obesity is that obesity is an “autoinflammatory” disease characterized by a chronic and low-grade inflammation [18,19], with several immune alterations including altered cell-mediated immune responses and leucocyte counts [20], principally in adipose tissue [21], where we have a localized inflammation [22]. Mechanisms beyond this are based on the links between obesity and both adipose tissue remodeling [23] and regulatory T cells [24]. Macrophage polarization [25], among other obesity-induced changes to macrophages [26], specifically due to adipocyte–macrophage interaction [27], are also involved within the inflammatory component of obesity.

The impacts obesity has on regeneration [9] could also explain, in part, such reduced antibody production due to the impaired regeneration immunity cells could have. Such observations would explain the reduced efficacy of vaccination in obese patients [28] as illustrated by the impaired immune response to influenza vaccination in obese humans [14] which could lead to recommend additional immunological stimulation (vaccination) for obese patients.

Exercise (combined or not with diet and/or pharmacological therapies) is among the most widely accepted approaches to controlling body weight and managing obesity [29,30,31]. Exercise has known benefits and effects on the immunity system [32,33] including antibodies [34], B lymphocytes [35], cytokines such as Interleukin-6 [36], antioxidant effects [37], regeneration adjuvants [38,39,40], and improved immunosurveillance and immunocompetence with an anti-inflammatory effects [41] via macrophage infiltration suppression [42]. Importantly, as illustrated above, the antibody-related immunity decline with obesity would be associated with the adiposity and its distribution rather than body weight [17]. This suggests that the benefits of exercise on antibodies for obese patients can be achieved even without weight loss, as illustrated by the reduced hepatic and visceral lipids following exercise training without weight loss [43]. The adiposity and fat distribution correlations, rather than body weight, with antibodies and immunity-related functions have been shown in other contexts such as inflammatory profiles [44,45] and IgG N-glycosylation [46]. Furthermore, central adiposity has been highlighted in correlation with other diseases [47,48] and health problems as well [49,50]. In addition, acute exercise (and therefore independent of weight lost) has a broad impact on immune functions, including granulocytosis, lymphocytosis (antibody-producing cells) and monocytosis [51], increased natural killer cells [52], which are very responsive to acute exercise [53], increased lymphokine-activated killer cells activity [54] and enhanced T cell activity [55]. Importantly, acute exercise might promote a redistribution in lymphocyte subsets [56] including B cells that produce the antibodies [57,58] and which are affected by obesity [59,60] via diverse pathways including leptin-induced reduction in B cells function [61] as well. These benefits reverse most of those induced by adiposity described earlier (Figure 1).

Such concepts indicate and support the importance of exercise even without weight loss so that an interrelation between exercise and immunity regulation has been described [62]. The absence of weight loss does not mean the absence of fat loss or fat redistribution. Indeed, with exercise, body composition can improve toward increased muscle development and/or a new fat distribution but without body weight loss. This pattern could explain the benefits of exercise that does not lead to weight loss, which is of a particular importance since among the anti-obesity therapies (diet, pharmacology, etc.), exercise represents the one with the ability to shift the body composition as well as fat distribution beyond weight loss [63,64]. Moreover, indirect weight-loss-independent benefits of exercise can improve immunity, for instance by reducing hypertension [65] that is associated with lower post COVID-19 vaccination antibodies titers [17].

The benefits of exercise in the context of obesity are well documented in the context of energy balance, glucose metabolism, adiposity, muscles development, cardiorespiratory fitness and lipids profile [66,67,68,69]. However, within this piece of writing, we also illustrate the beneficial effects of exercise on obesity from an immunological perspective that focuses on antibodies. The interesting point is that the exercise effects are seen even with the absence of body weight loss. Therefore, this indicates that a focus on adiposity loss and fat distribution patterns [70] should replace the use of body weight as a medical parameter which correlates with the need to further focus, for instance, on waist circumference, which reflects to some extent visceral obesity, in clinical practice [71]. The concept of fat distribution and adiposity vs. overweight would also explain the concept “metabolically healthy obesity” [72,73], defined by body mass index that could lead to the concept of “immunologically healthy obesity”.

We hope our work could represent an additional encouragement of physical activity and a healthy diet towards a better lifestyle for obese patients even if it does not necessarily lead to weight loss, especially that the benefits shown without weight loss are various and include decreased circulating interleukin-6 [74], reduced hepatic and visceral lipids [43], increased insulin sensitivity [75] and improved endothelium-dependent vasodilation [76]. The possible application of such concepts would be the prescription of exercise to improve the antibody properties of obese patients even if it does not lead to weight loss since, for the COVID-19 mRNA vaccine for instance, low antibody titers have been associated with a higher waist circumference rather than high body weights [17], suggesting, once more, that the impact would be due to the fat distribution (central vs. peripheral obesity) [4,10] rather than increased body weight or even body fat percentage. Indeed, exercise can impact the body composition and fat distribution independently from body weight. This area of interaction between adiposity, fat distribution and immunology is worth further exploring in diverse contexts to develop new therapies, optimize the existing treatments and increase the awareness of how important weight-loss-independent effects of exercise are.

## Figures and Tables

**Figure 1:**
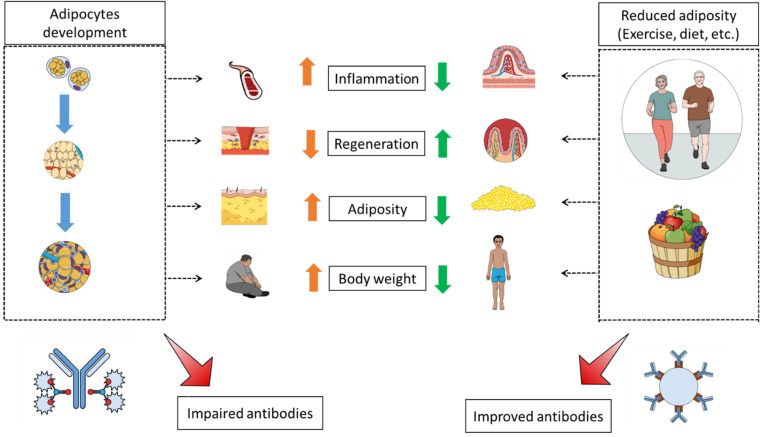
**Antibody patterns and immunity performance between increased adiposity and exercise.** Immunity functions and antibody-related patterns such as inflammation and regeneration are negatively impacted by adiposity development but corrected/improved by exercise and other adiposity-reducing approaches.

## Data Availability

Not applicable.
